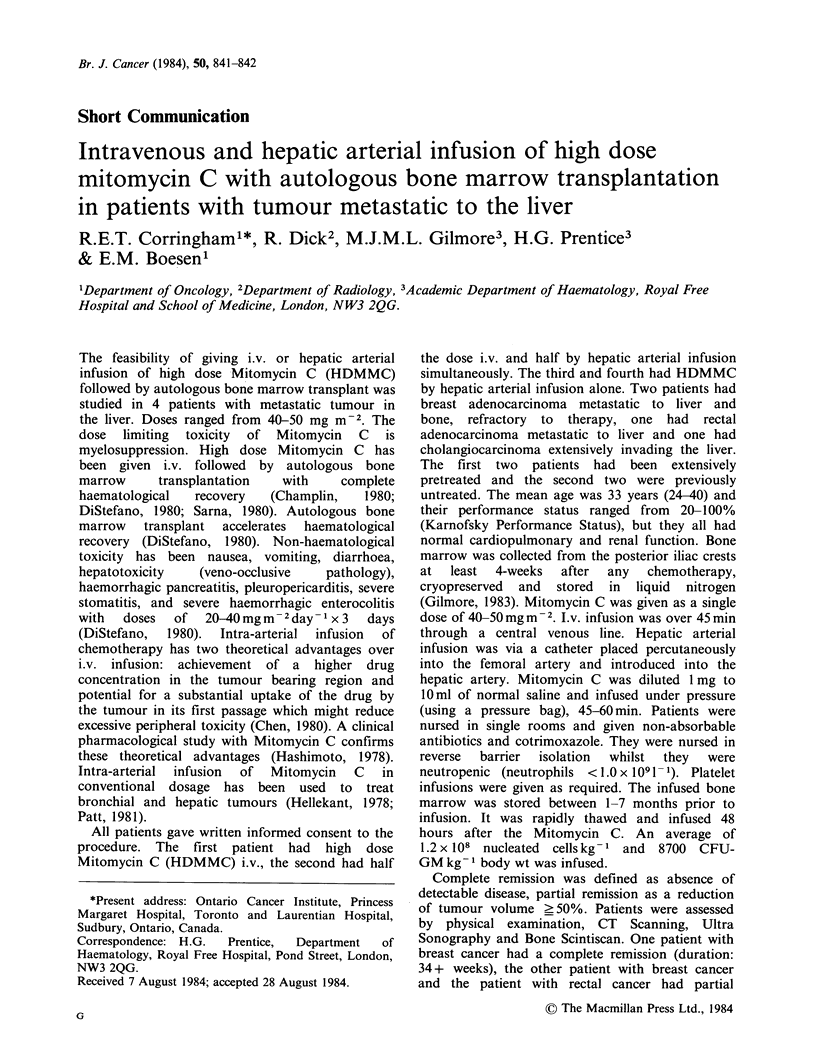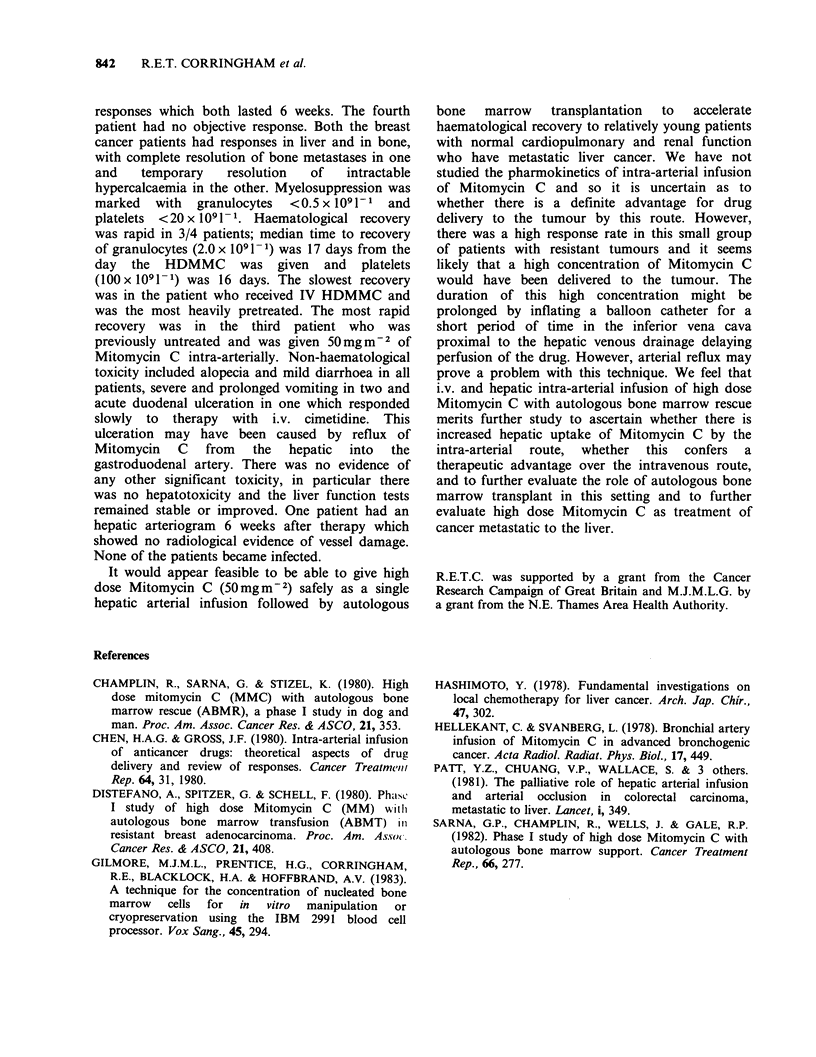# Intravenous and hepatic arterial infusion of high dose mitomycin C with autologous bone marrow transplantation in patients with tumour metastatic to the liver.

**DOI:** 10.1038/bjc.1984.265

**Published:** 1984-12

**Authors:** R. E. Corringham, R. Dick, M. J. Gilmore, H. G. Prentice, E. M. Boesen


*Br. J. Cancer* (1984), **50**, 841–842

**Short Communication**

### Intravenous and hepatic arterial infusion of high dose mitomycin C with autologous bone marrow transplantation in patients with tumour metastatic to the liver

R.E.T. Corringham[1]*, R. Dick[2], M.J.M.L. Gilmore[3], H.G. Prentice[3] & E.M. Boesen[1]

[1]*Department of Oncology,* [2]*Department of Radiology,* [3]*Academic Department of Haematology, Royal Free Hospital and School of Medicine, London, NW3 2QG.*

The feasibility of giving i.v. or hepatic arterial infusion of high dose Mitomycin C (HDMMC) followed by autologous bone marrow transplant was studied in 4 patients with metastatic tumour in the liver. Doses ranged from 40–50 mg $m^{-2}$. The dose limiting toxicity of Mitomycin C is myelosuppression. High dose Mitomycin C has been given i.v. followed by autologous bone marrow transplantation with complete haematological recovery (Champlin, 1980; DiStefano, 1980; Sarna, 1980). Autologous bone marrow transplant accelerates haematological recovery (DiStefano, 1980). Non-haematological toxicity has been nausea, vomiting, diarrhoea, hepatotoxicity (veno-occlusive pathology), haemorrhagic pancreatitis, pleuropericarditis, severe stomatitis, and severe haemorrhagic enterocolitis with doses of 20–40 mg $m^{-2}$ $day^{-1}$ × 3 days (DiStefano, 1980). Intra-arterial infusion of chemotherapy has two theoretical advantages over i.v. infusion: achievement of a higher drug concentration in the tumour bearing region and potential for a substantial uptake of the drug by the tumour in its first passage which might reduce excessive peripheral toxicity (Chen, 1980). A clinical pharmacological study with Mitomycin C confirms these theoretical advantages (Hashimoto, 1978). Intra-arterial infusion of Mitomycin C in conventional dosage has been used to treat bronchial and hepatic tumours (Hellekant, 1978; Patt, 1981).

All patients gave written informed consent to the procedure. The first patient had high dose Mitomycin C (HDMMC) i.v., the second had half the dose i.v. and half by hepatic arterial infusion simultaneously. The third and fourth had HDMMC by hepatic arterial infusion alone. Two patients had breast adenocarcinoma metastatic to liver and bone, refractory to therapy, one had rectal adenocarcinoma metastatic to liver and one had cholangiocarcinoma extensively invading the liver. The first two patients had been extensively pretreated and the second two were previously untreated. The mean age was 33 years (24–40) and their performance status ranged from 20–100% (Karnofsky Performance Status), but they all had normal cardiopulmonary and renal function. Bone marrow was collected from the posterior iliac crests at least 4-weeks after any chemotherapy, cryopreserved and stored in liquid nitrogen (Gilmore, 1983). Mitomycin C was given as a single dose of 40–50 mg $m^{-2}$. I.v. infusion was over 45 min through a central venous line. Hepatic arterial infusion was via a catheter placed percutaneously into the femoral artery and introduced into the hepatic artery. Mitomycin C was diluted 1 mg to 10 ml of normal saline and infused under pressure (using a pressure bag), 45–60 min. Patients were nursed in single rooms and given non-absorbable antibiotics and cotrimoxazole. They were nursed in reverse barrier isolation whilst they were neutropenic (neutrophils $< 1.0 \times 10^9 l^{-1}$). Platelet infusions were given as required. The infused bone marrow was stored between 1–7 months prior to infusion. It was rapidly thawed and infused 48 hours after the Mitomycin C. An average of $1.2 \times 10^8$ nucleated cells $kg^{-1}$ and 8700 CFU-GM $kg^{-1}$ body wt was infused.

Complete remission was defined as absence of detectable disease, partial remission as a reduction of tumour volume $\geq 50\%$. Patients were assessed by physical examination, CT Scanning, Ultra Sonography and Bone Scintiscan. One patient with breast cancer had a complete remission (duration: 34+ weeks), the other patient with breast cancer and the patient with rectal cancer had partial

*Present address: Ontario Cancer Institute, Princess Margaret Hospital, Toronto and Laurentian Hospital, Sudbury, Ontario, Canada.
Correspondence: H.G. Prentice, Department of Haematology, Royal Free Hospital, Pond Street, London, NW3 2QG.
Received 7 August 1984; accepted 28 August 1984.

© The Macmillan Press Ltd., 1984

responses which both lasted 6 weeks. The fourth patient had no objective response. Both the breast cancer patients had responses in liver and in bone, with complete resolution of bone metastases in one and temporary resolution of intractable hypercalcaemia in the other. Myelosuppression was marked with granulocytes $<0.5\times10^9\,l^{-1}$ and platelets $<20\times10^9\,l^{-1}$. Haematological recovery was rapid in 3/4 patients; median time to recovery of granulocytes ($2.0\times10^9\,l^{-1}$) was 17 days from the day the HDMMC was given and platelets ($100\times10^9\,l^{-1}$) was 16 days. The slowest recovery was in the patient who received IV HDMMC and was the most heavily pretreated. The most rapid recovery was in the third patient who was previously untreated and was given 50 mg m$^{-2}$ of Mitomycin C intra-arterially. Non-haematological toxicity included alopecia and mild diarrhoea in all patients, severe and prolonged vomiting in two and acute duodenal ulceration in one which responded slowly to therapy with i.v. cimetidine. This ulceration may have been caused by reflux of Mitomycin C from the hepatic into the gastroduodenal artery. There was no evidence of any other significant toxicity, in particular there was no hepatotoxicity and the liver function tests remained stable or improved. One patient had an hepatic arteriogram 6 weeks after therapy which showed no radiological evidence of vessel damage. None of the patients became infected.

It would appear feasible to be able to give high dose Mitomycin C (50 mg m$^{-2}$) safely as a single hepatic arterial infusion followed by autologous bone marrow transplantation to accelerate haematological recovery to relatively young patients with normal cardiopulmonary and renal function who have metastatic liver cancer. We have not studied the pharmokinetics of intra-arterial infusion of Mitomycin C and so it is uncertain as to whether there is a definite advantage for drug delivery to the tumour by this route. However, there was a high response rate in this small group of patients with resistant tumours and it seems likely that a high concentration of Mitomycin C would have been delivered to the tumour. The duration of this high concentration might be prolonged by inflating a balloon catheter for a short period of time in the inferior vena cava proximal to the hepatic venous drainage delaying perfusion of the drug. However, arterial reflux may prove a problem with this technique. We feel that i.v. and hepatic intra-arterial infusion of high dose Mitomycin C with autologous bone marrow rescue merits further study to ascertain whether there is increased hepatic uptake of Mitomycin C by the intra-arterial route, whether this confers a therapeutic advantage over the intravenous route, and to further evaluate the role of autologous bone marrow transplant in this setting and to further evaluate high dose Mitomycin C as treatment of cancer metastatic to the liver.

R.E.T.C. was supported by a grant from the Cancer Research Campaign of Great Britain and M.J.M.L.G. by a grant from the N.E. Thames Area Health Authority.

### References

CHAMPLIN, R., SARNA, G. & STIZEL, K. (1980). High dose mitomycin C (MMC) with autologous bone marrow rescue (ABMR), a phase I study in dog and man. *Proc. Am. Assoc. Cancer Res. & ASCO*, **21**, 353.

CHEN, H.A.G. & GROSS, J.F. (1980). Intra-arterial infusion of anticancer drugs: theoretical aspects of drug delivery and review of responses. *Cancer Treatment Rep.* **64**, 31, 1980.

DISTEFANO, A., SPITZER, G. & SCHELL, F. (1980). Phase I study of high dose Mitomycin C (MM) with autologous bone marrow transfusion (ABMT) in resistant breast adenocarcinoma. *Proc. Am. Assoc. Cancer Res. & ASCO*, **21**, 408.

GILMORE, M.J.M.L., PRENTICE, H.G., CORRINGHAM, R.E., BLACKLOCK, H.A. & HOFFBRAND, A.V. (1983). A technique for the concentration of nucleated bone marrow cells for *in vitro* manipulation or cryopreservation using the IBM 2991 blood cell processor. *Vox Sang.*, **45**, 294.

HASHIMOTO, Y. (1978). Fundamental investigations on local chemotherapy for liver cancer. *Arch. Jap. Chir.*, **47**, 302.

HELLEKANT, C. & SVANBERG, L. (1978). Bronchial artery infusion of Mitomycin C in advanced bronchogenic cancer. *Acta Radiol. Radiat. Phys. Biol.*, **17**, 449.

PATT, Y.Z., CHUANG, V.P., WALLACE, S. & 3 others. (1981). The palliative role of hepatic arterial infusion and arterial occlusion in colorectal carcinoma, metastatic to liver. *Lancet*, **i**, 349.

SARNA, G.P., CHAMPLIN, R., WELLS, J. & GALE, R.P. (1982). Phase I study of high dose Mitomycin C with autologous bone marrow support. *Cancer Treatment Rep.*, **66**, 277.